# The Role of Receptor for Advanced Glycation End Products in Airway Inflammation in CF and CF related Diabetes

**DOI:** 10.1038/srep08931

**Published:** 2015-03-10

**Authors:** Siobhain Mulrennan, Svetlana Baltic, Shashi Aggarwal, Jamie Wood, Alina Miranda, Felicity Frost, Joey Kaye, Philip J. Thompson

**Affiliations:** 1 Department of Respiratory Medicine, Sir Charles Gairdner Hospital, Nedlands, Western Australia; 2 Lung Institute of Western Australia, Nedlands, Western Australia; 3 Centre for Asthma, Allergy and Respiratory Research, School of Medicine and Pharmacology, University of Western Australia, Western Australia; 4 PathWest, Sir Charles Gairdner Hospital, Nedlands, Western Australia; 5 Department of Endocrinology, Sir Charles Gairdner Hospital, Nedlands, Western Australia

## Abstract

Cystic Fibrosis (CF) is often accompanied by diabetes leading to worsening lung function, the reason for which is unclear. The receptor for advanced-glycation-end-products (RAGE) regulates immune responses and inflammation and has been linked to diabetes and possibly CF. We performed a pilot study to determine if CF and CF-related diabetes (CFRD) are associated with enhanced RAGE expression. Full length (fl)RAGE, soluble (s)RAGE, endogenous soluble (es)RAGE, S100A12 (enRAGE) and advanced-glycation-end-products (AGE) expression was assessed in serum, white blood cells and sputum of patients with CF; diabetes; CFRD and healthy subjects. Sputum enRAGE/sRAGE ratios were high in CF but particularly in CFRD which negatively correlated with % predicted FEV1. Serum AGE and AGE/sRAGE ratios were high in diabetics but not in CF. A complex, multifaceted approach was used to assess the role of RAGE and its ligands which is fundamental to determining their impact on airway inflammation. There is a clear association between RAGE activity in the airways of CF and CFRD patients that is not evident in the vascular compartment and correlates with lung function, in contrast to diabetes. This strongly suggests a role for RAGE in contributing to the inflammatory overdrive seen in CF and to a greater extent in CFRD.

Cystic fibrosis (CF) is the most common autosomal recessive condition in Caucasians and is associated with bronchiectasis, airway inflammation and an increase in diabetes^1^. Cystic Fibrosis Related Diabetes (CFRD) prevalence increases with age; with more than 50% of CF patients having diabetes by age 40. CFRD patients have a higher mortality than CF alone and there is a strong association between CFRD and deterioration in lung function and clinical status^2^. Why diabetes should do this is unknown.

The receptor for advanced glycation end products (RAGE) is a member of the immunoglobulin superfamily of cell surface molecules. The receptor is membrane bound and is also known as full length (fl)RAGE or membrane RAGE (mRAGE). It is a multi-ligand receptor and regarded as a central mediator in chronic inflammatory and immune responses^3^. RAGE is found in human airways with high basal levels of RAGE expressed in pulmonary tissue^4^. It is also found on pro-inflammatory and immuno-competent cells such as neutrophils, monocytes, macrophages, and T and B lymphocytes^3^,^5^.

RAGE binds a broad range of ligands associated with inflammatory responses, including advanced glycation end products (AGE), β-sheet fibrillary structures (β-amyloid & serum amyloid A), amphoterin (HMGB1) and members of the S100/calgranulin family (such as S100A12 also known as enRAGE)^3^,^5^,^6^. EnRAGE is secreted by activated granulocytes and is a marker of inflammation in CF^7^. These ligands, as well as activating RAGE, also up-regulate RAGE production and thereby perpetuate the inflammatory response due to downstream production of cytokines, adhesion molecules and matrix metalloproteinases^5^.

RAGE expression and its signaling are regulated both by its ligands and by RAGE isoforms known collectively as soluble RAGE (sRAGE). sRAGE contains the extracellular domain of RAGE and can bind to circulating pro-inflammatory ligands preventing their binding to mRAGE thereby preventing RAGE activation. sRAGE consists of a combination of isoforms that are generated in two distinct ways: 1) cleaved RAGE (cRAGE) which results from the proteolytic cleavage of mRAGE (ectodomain shedding) from the cell membrane; and 2) alternative splicing of the RAGE transcript resulting in 10 variants detected in the human lung^8^,^9^. Of these the most significant is an endogenous soluble RAGE (esRAGE). Importantly decreased levels of esRAGE and/or increases in mRAGE are thought to enhance RAGE mediated inflammation^10^.

In healthy adults, serum levels of sRAGE and RAGE ligands are well matched^11^. In contrast decreased levels of serum sRAGE is linked to atherosclerosis, arthritis and CF^6^,^12^,^13^. Levels are lower in diabetic patients with complications versus those without^3^. Diabetes is also associated with raised AGE levels and subsequent up regulation of inflammation occurs as a consequence of AGE activation of RAGE^3^.

The link between RAGE and CF has been reported in only a small number of studies. CF airway neutrophils have increased RAGE compared to peripheral blood neutrophils while CF airway fluid has increased levels of enRAGE and lacks sRAGE the protective decoy receptor^6^,^7^. The association between RAGE, inflammation, the lung and diabetes suggests that RAGE may have an important role in CF but particularly in CFRD.

We hypothesized that chronic RAGE activation and resulting increased airway inflammation leads to worsening lung function and clinical status in CFRD compared to non-diabetic CF patients. To address this we quantified RAGE expression in induced sputum, peripheral blood leukocytes (PBLs) and in serum. sRAGE, esRAGE, fl/mRAGE, the ligands enRAGE (S100A12) and AGE, were assessed in CFRD, CF, Diabetics and healthy subjects. We explored the interaction of the various elements of the RAGE pathway and correlated this with lung function.

## Results

### Clinical parameters

All patients were categorized into one of four groups: CF, Diabetes (type 1, insulin dependent), CFRD or healthy controls and their diagnoses confirmed by a specialist physician. The subject clinical characteristics are provided in Table 1. Five patients in each group were sequentially selected for sputum analysis.

### Induced sputum cell counts

Sputum differential cell counts in CF and CFRD subjects showed predominantly neutrophils (high cellularity), while healthy subjects had predominantly macrophages (low cellularity) and diabetics mixed macrophages/neutrophils (low cellularity).

### mRNA expression of RAGE in peripheral blood leukocytes and in sputum

**Figure 1:**
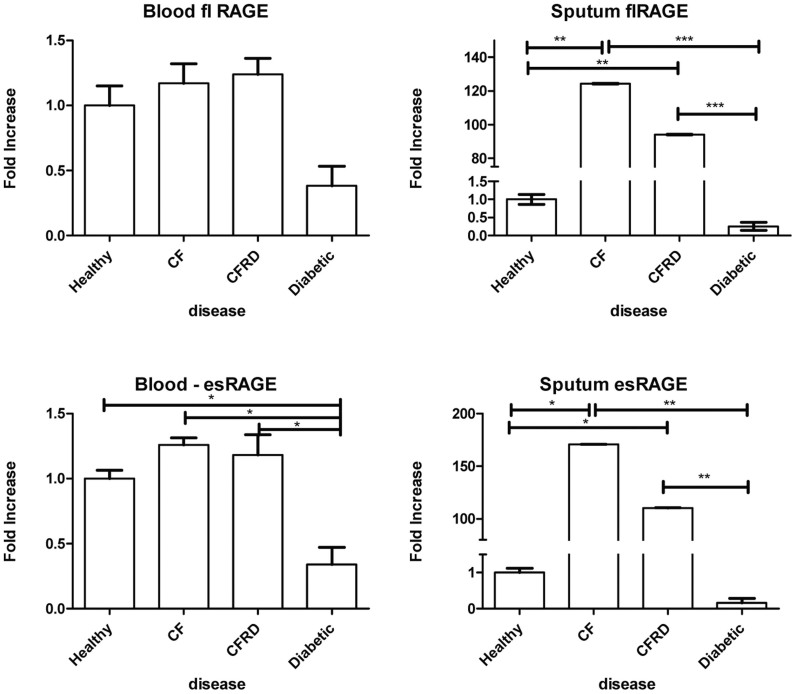
flRAGE and esRAGE mRNA Expression in blood and sputum. The mRNA expression of full length (fl)RAGE and endogenous soluble (es)RAGE was evaluated by RT-qPCR in total RNA isolated from peripheral blood leucocytes and sputum. Results are shown as Fold increase representing a relative expression according to the levels of expression in healthy controls. (n ≥ 5); * p < 0.05; **p < 0.01; ***< 0.001.

The mRNA expression of fl/mRAGE and esRAGE (splice variant) in PBLs of healthy controls, CF and CFRD patients were similar, while it was down-regulated in diabetic patients (esRAGE p < 0.05)(Fig. 1).

In sputum, the mRNA for fl/mRAGE and esRAGE were markedly overexpressed in CF compared to both healthy individuals (p < 0.01 for fl/mRAGE; p < 0.05 for esRAGE) and diabetics (p < 0.001 for fl/mRAGE; p < 0.01 for esRAGE). Down-regulation of both RAGE isoforms at the mRNA level was observed in diabetes compared to healthy individuals, (p > 0.05). fl/mRAGE and esRAGE mRNA were overexpressed in CFRD patients to a lesser extent than in the in sputums of CF only group (p > 0.05) (Fig. 1).

### RAGE immunocytochemistry

**Figure 2:**
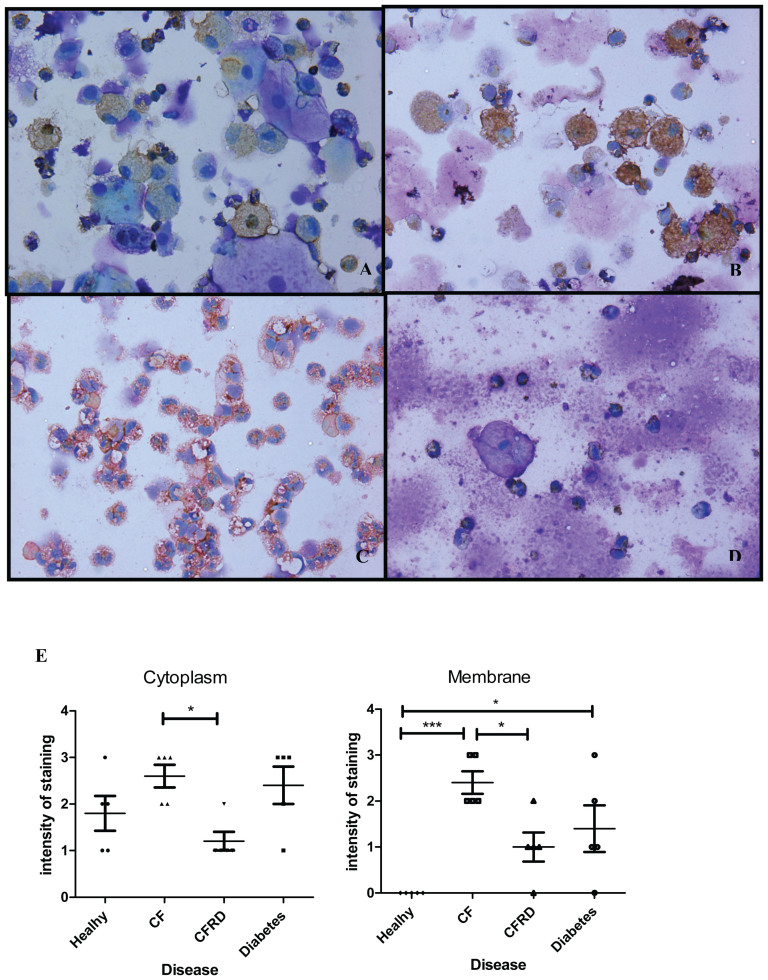
flRAGE protein expression in sputum samples from A: Healthy individuals, B: Diabetic subjects, C: cystic fibrosis (CF) and D: CF Related Diabetic (CFRD) subjects. The immunohistochemical expression is identified predominantly in macrophages in healthy and in diabetic subjects (A and B), while full lenght (fl)RAGE expression is predominantly identified within the cytoplasm of neutrophils in CF and CFRD subjects (C and D). Graph represents total intensity of cell staining in the cytoplasm and membranes in healthy, CF, CFRD and diabetic patients assessed according to Remmele's sliding scale (E). * p < 0.05; **p < 0.01; ***< 0.001.

Fl/mRAGE immunocytochemistry of sputum showed expression in a range of cell types. Healthy individuals showed fl/mRAGE predominantly in macrophages with strong granular diffuse cytoplasmic staining, while no membrane staining was seen (Fig. 2). Diabetics had fl/mRAGE expression in macrophages, neutrophils and lymphocytes. Cytoplasmic staining was similar to healthy patients, but membrane staining was significantly higher in diabetics than in healthy subjects (p < 0.05; Fig. 2E). In CF fl/mRAGE expression on neutrophils was evident in 5/5 subjects and in 4/5 CFRD subjects. For both CF and CFRD patients, neutrophil fl/mRAGE expression was apparent on the membranes and diffuse in cytoplasm. However, the CFRD group had significantly less fl/mRAGE staining in the cytoplasm and cell membranes than the CF alone group (p < 0.05). A small number of eosinophils and lymphocytes were observed in the CF subjects and these showed fl/mRAGE expression; and this was localized predominantly to the cell membrane (Fig. 2). When all cells were assessed collectively, cell-membrane staining in CF patients was significantly higher than in healthy (p < 0.001), diabetic (p < 0.05) and CFRD patients (p < 0.05).

### sRAGE, AGE and enRAGE Protein Expression in sputum and serum

**Figure 3:**
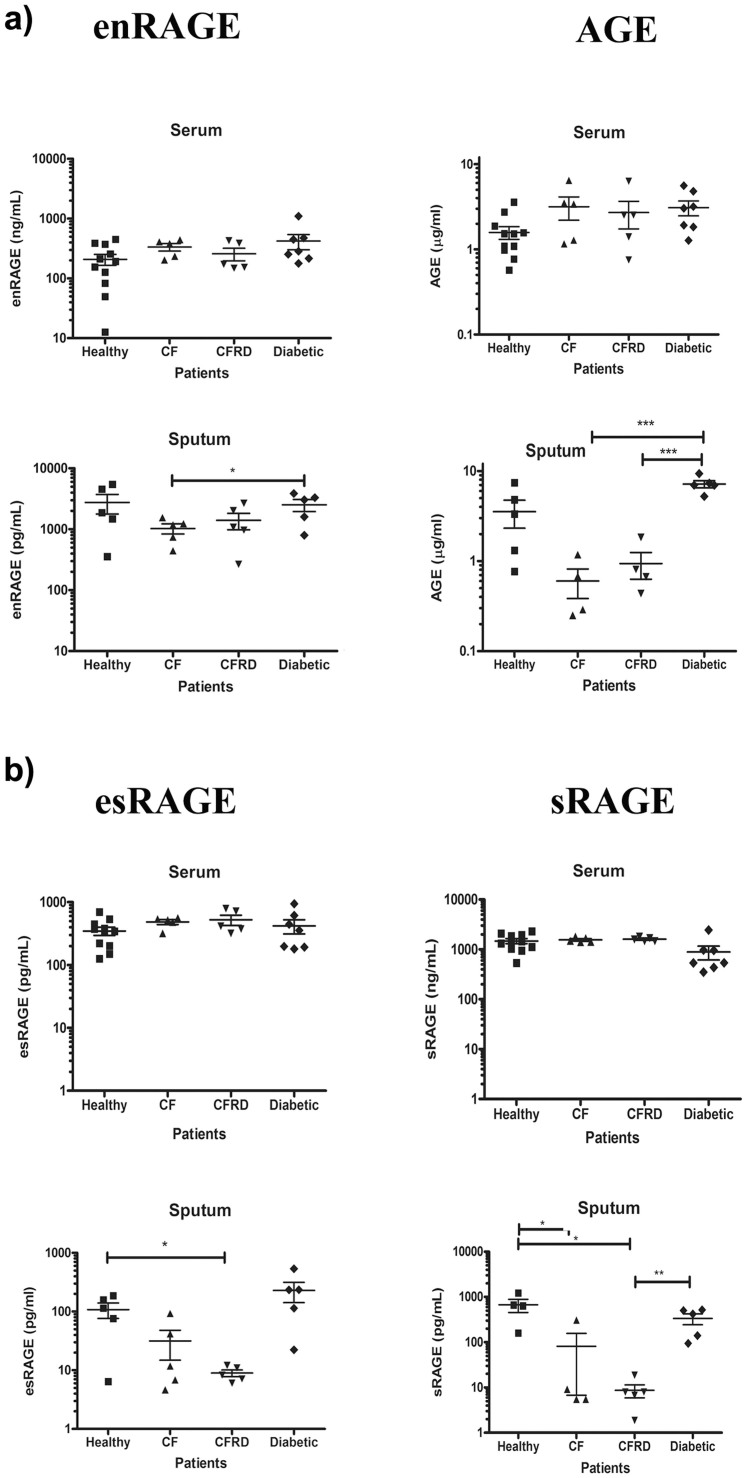
Expression of RAGE isoforms and its ligands in serum and sputum a) Expression of RAGE ligands in serum and sputum.The levels of enRAGE and AGE were assessed using ELISA. b). Expression of soluble RAGE isoforms in serum and sputum. The levels of endogenous soluble (es)RAGE and soluble (s)RAGE were assessed using ELISA in duplicates. The line in the scatter plots indicates the Mean ± SEM for each assay. (n ≥ 5). * p < 0.05; **p < 0.01; ***< 0.001.

**Figure 4:**
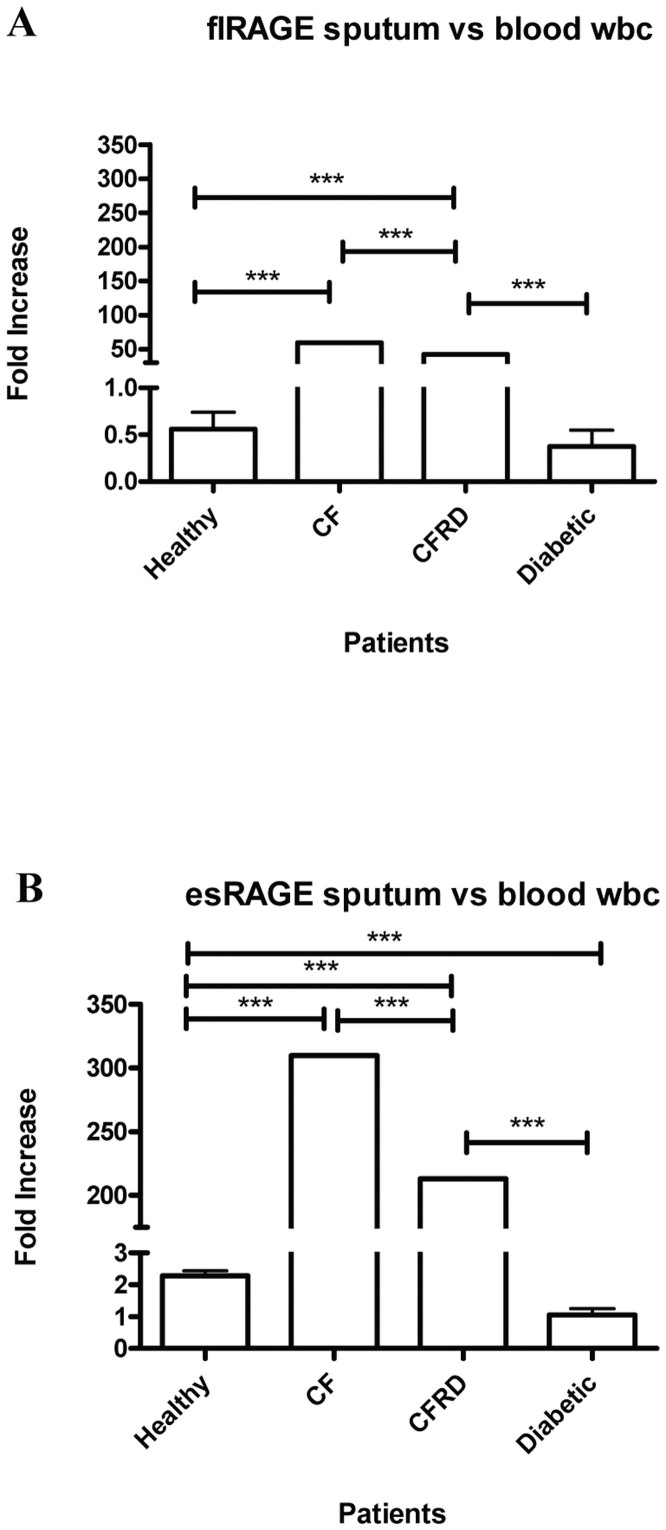
The expression of flRAGE and esRAGE mRNA in sputum and peripheral blood white blood cells. Expression of the full length (fl)RAGE (A) and endogenous soluble (es)RAGE (B) mRNA was assessed by one step RT-qPCR. Their expression in the sputum was compared to the levels measured in peripheral blood peripheral blood leucocytes within the same patient group (n ≥ 5). * p < 0.05; **p < 0.01; ***< 0.001.

sRAGE protein levels, as well as RAGE ligands (AGE and enRAGE) were measured in sputum and in serum. Not surprisingly serum sRAGE, esRAGE, AGE and enRAGE did not differ significantly among any disease group or healthy patients (Fig. 3a and 4b).

Sputum AGE concentrations were higher in diabetics than in healthy subjects (p < 0.05), CF (p < 0.001) and CFRD patients (p < 0.001) (Fig. 3a). Although sputum enRAGE concentrations in CF and CFRD patients were decreased compared to healthy and diabetic patients there was only a statistical difference evident for CF versus diabetics (p < 0.05) (Fig. 3a).

esRAGE concentrations were lower in CFRD sputa than in healthy (p < 0.05) and diabetic samples (p < 0.05) (Fig. 3b). Sputum sRAGE concentrations were significantly lower in CF and CFRD patients compared to healthy subjects (p < 0.05 & p < 0.01, respectively) while CFRD sRAGE sputum concentrations were also significantly lower than in diabetics (p < 0.01) (Fig. 3b).

### Characterisation of RAGE mRNA expression in disease states

**Figure 5:**
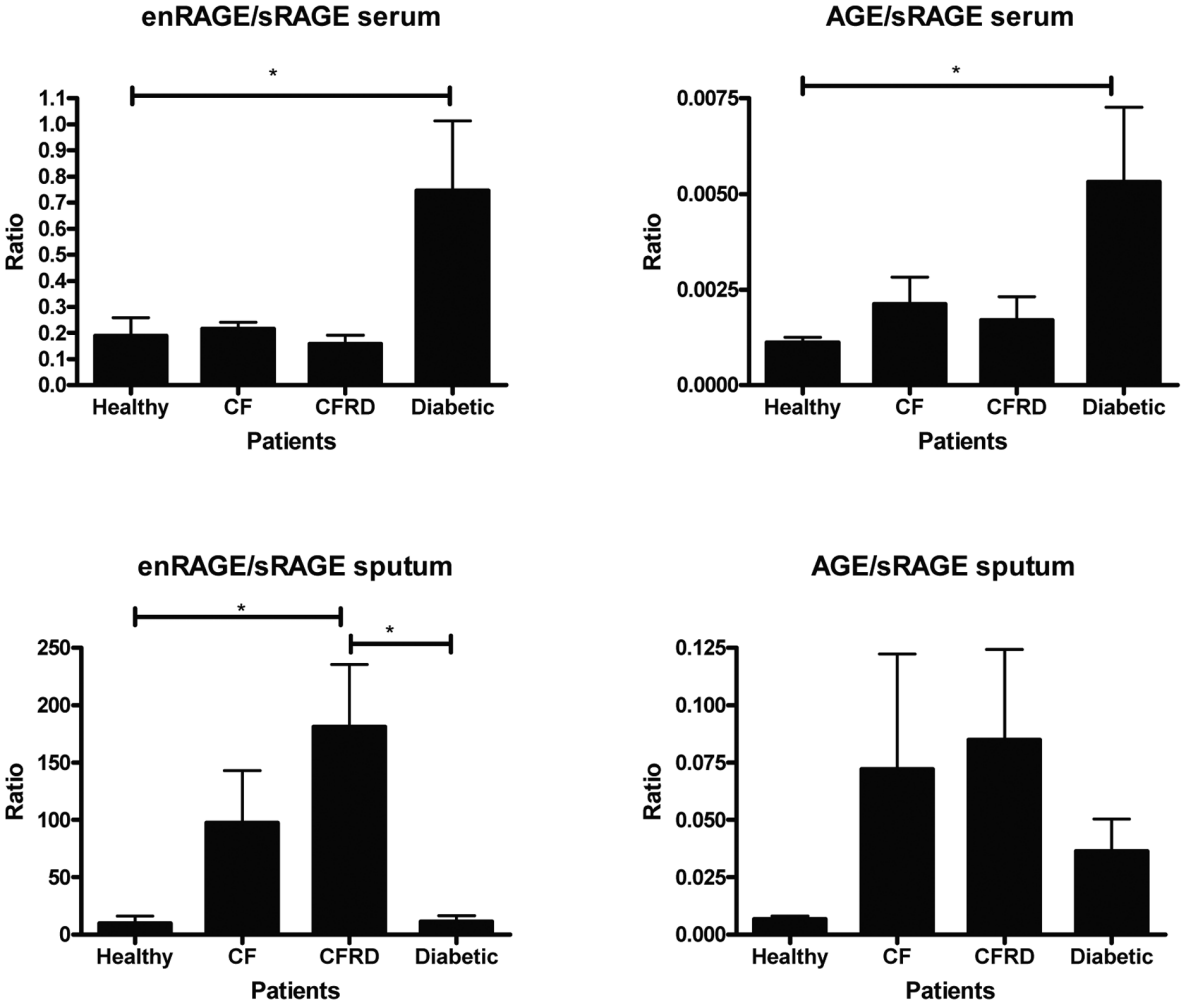
The ratio of AGE/enRAGE and sRAGE in serum and sputum. The levels of soluble (s)RAGE, enRAGE and advance glycation end products (AGE) were assessed in serum and sputum by ELISA. The ratio of enRAGE/sRAGE and AGE/sRAGE was calculated for serum and sputum for each patient and grouped based on disease status. (n = 5). * p < 0.05; **p < 0.01; ***< 0.001.

The magnitude of fl/mRAGE and esRAGE mRNA expression varied in different tissues. To assess whether serum markers reflect airway inflammation, RAGE expression in sputum and serum/PBLs were compared. Differences in fl/mRAGE and esRAGE mRNA expression were observed for PBLs versus induced sputum cells within the same subjects. In healthy patients there was less fl/mRAGE and twice as much esRAGE mRNA in the sputum than in PBLs (p < 0.001 for both measurements). In CF alone patients, however, both fl/mRAGE and esRAGE mRNA expression was much greater in sputum cells than in PBLs (p < 0.001 for both). This increase was of a different magnitude with esRAGE mRNA level being 6-fold greater than for flRAGE levels (Fig. 4). In CFRD the increase in esRAGE expression in sputum v PBLs was of lower magnitude than in CF patients (300X versus 200X, p < 0.001) (Fig. 5). In Diabetic patients, esRAGE mRNA expression was similar in sputum and PBLs, but they had significantly less fl/mRAGE mRNA expression in sputum compared to PBLs (p < 0.001) (Fig. 4).

### The Ratio of Protein expression of AGE and enRAGE to sRAGE

The ratios of protein expression of pro-inflammatory AGE and enRAGE and the presumed protective sRAGE were assessed in each study group. In serum, this ratio was only significantly greater for diabetics (for both AGE and enRAGE) and is in keeping with diabetes being a systemic vascular orientated disease. However in sputum this ratio was significantly greater for CFRD (enRAGE only) highlighting the localization of inflammation to the airways in this disease group (Fig. 5).

### Analysis of interaction between sRAGE, AGE, flRAGE in Sputum

**Figure 6:**
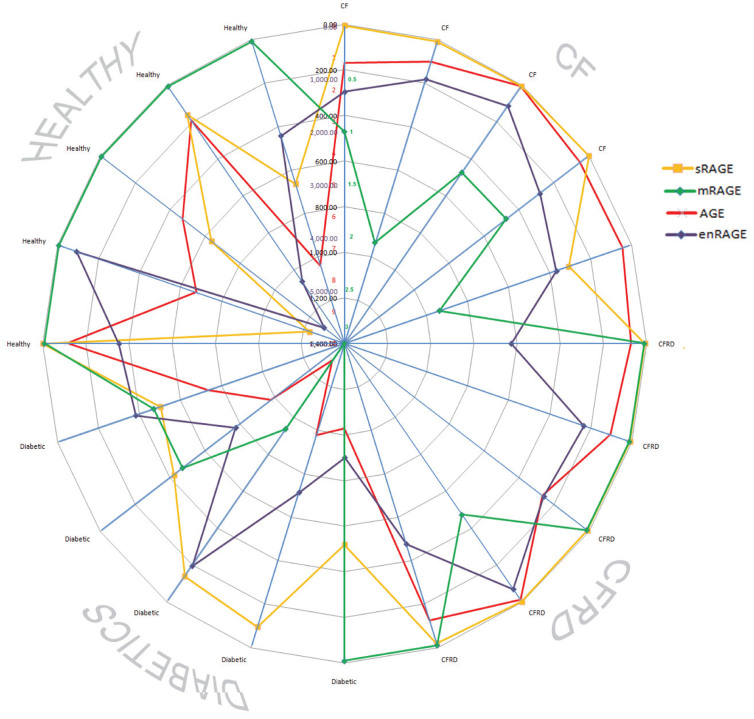
Expression of membrane flRAGE (mRAGE), sRAGE, AGE and enRAGE in induced sputum of patients in all disease groups. Radar graph displays levels of membrane-bound (m)RAGE, soluble (s)RAGE, advance glycation end products (AGE) and enRAGE expression for each patient in Healthy, CF, CFRD and Diabetes group. Each mediator is presented on the appropriate scale; mRAGE (0-3, green line and numbers) enRAGE (0-6000, blue line and numbers); AGE (0-10, red line and numbers) and sRAGE (0-1400, yellow line, black numbers), with highest numbers being in the centre of the graph.

All of the assessed mediators/factors in sputum samples were compared and there were marked differences between all patient groups. CF patients were are all characterised by low sRAGE and AGE levels. Interestingly, patients with diabetes demonstrated relatively high levels of mRAGE but equally sRAGE and AGE were also significantly up-regulated. In CFRD all four variables appeared to be elevated (Fig. 6).

### Correlation between AGE, enRAGE, esRAGE and %predicted FEV1

**Figure 7:**
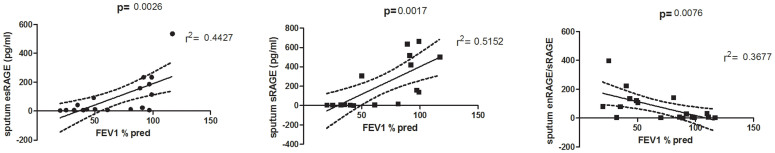
Correlation between FEV1%pred and mediators in sputum. Graph represents the correlation between FEV1%predicted and a) endogenous soluble (es)RAGE b) soluble (s)RAGE and c) ratio of enRAGE/sRAGE in sputum of patients.

To assess the degree to which the various RAGE system components may be contributing to lung function all patient data for sRAGE, esRAGE and the ratio enRAGE/sRAGE were correlated with FEV1 as a percentage of predicted value. This was significantly positively correlated with sRAGE and esRAGE (p = 0.0017; p = 0.0026) and negatively correlated with the ratio of enRAGE/sRAGE (p = 0.0076) (Fig. 7).

## Discussion

CF is characterised by persistent airway inflammation and bronchiectasis. Individuals with CF are susceptible to developing diabetes and accelerated respiratory decline, but the mechanism is unclear. RAGE and RAGE ligands are linked to chronic inflammatory states and we hypothesised that collectively they contribute to the rapid decline in lung function seen in CFRD. In this study we have demonstrated that the balance between protective sRAGE and pro-inflammatory RAGE ligands is adversely affected in patients with CF and considerably more so in CFRD and is potentially associated with worse lung function. Evaluating the balance between protective sRAGE, fl/mRAGE and RAGE ligands to determine the impact on inflammation seemed fundamental while peripheral blood assessments were likely to be relatively insensitive to the role of RAGE in airway diseases compared to diabetes.

This is the first study to characterize differences in expression of RAGE and its ligands in both serum and sputum in CF and diabetes. Although CF patients failed to show any difference in serum ligand/RAGE expression (compared to normals) they did have marked up-regulation of fl/mRAGE, down-regulation of sRAGE and high AGE/sRAGE ratios in sputa. The CFRD patients differed with the non-diabetic CF patients in several aspects. They had less fl/mRAGE detected in sputum cells and had a further reduction in protective sRAGE. Most importantly, in CFRD the ratio of enRAGE/sRAGE in sputum was considerably higher than in any other patient group making them more vulnerable to increased inflammatory insult. For diabetic patients there was a much higher proportion of serum enRAGE (and AGE) than protective sRAGE compared to healthy controls. This ratio strikingly differentiated diabetic patients from the other disease groups and importantly healthy subjects.

The serum level of circulating sRAGE in healthy subjects was similar to that previously reported^14^. Interestingly, the serum sRAGE and esRAGE levels did not differ significantly between patient groups. Low serum levels of sRAGE have been reported to be associated with a number of human diseases including diabetes^6^,^15^. Plasma sRAGE has been promoted as a biomarker of acute lung injury and acute pulmonary inflammatory response^16^. However in our study of more chronic airway diseases serum sRAGE is not sensitive to disease activity. Understandably in acute settings initial levels of sRAGE may be increased, while in chronic disease it may revert to being normal. RAGE ligands, AGE and enRAGE, are increased in the plasma/serum in various diseases, including CF and diabetes^6^,^7^,^17^. However, in this study, although serum enRAGE from diabetic patients was increased substantially it did not differ from healthy subjects statistically, possibly due to the small sample size. Increased serum RAGE was not seen in CF and CFRD patients, indicating a need to reassess whether changes in serum RAGE levels are informative in chronic airway inflammatory diseases.

CF and CFDR patients differed significantly from controls and diabetics with respect to both sputum RAGE and ligand concentrations. In particular fl/mRAGE as well as the ratio between pro-inflammatory enRAGE and protective sRAGE were increased. sRAGE and esRAGE were significantly lower in CF and CFRD patient sputa, than in healthy controls while CFRD patients had much lower levels than diabetics. The sRAGE levels in CF sputa were lower than previously reported^6^. However in that study, only 2/20 patients had detectable sRAGE levels while in this study sRAGE was detected in 9/10 CF patients and in 4/5 healthy subjects. This increased detection rate probably reflects differences in induced sputum protocols and assay conditions. sRAGE is thought to be protective and corresponds to the extracellular domain of full length RAGE^18^. Thus despite an overexpression of mRNA for esRAGE in sputa, the protein concentrations of sRAGE and esRAGE were lower in CFRD and CF patients compared to controls. Although this might reflect alteration to post transcriptional or translational regulation, it is more likely due to increased binding to ligands and also explains the low AGE levels seen. Thus the lower levels of sRAGE and esRAGE (acting as inhibitory decoy receptors) may indirectly contribute to inflammation seen in CF/CFRD by facilitating ligand binding to fl/mRAGE. The concept of sRAGE being protective is supported by increased sRAGE and decreased enRAGE/sRAGE sputum ratios correlating with %predicted FEV1. Furthermore, the percentage of sputa cells expressing fl/mRAGE and the magnitude of expression was highest in CF and CFRD patients and was predominantly in neutrophils. Since the antibodies used cannot differentiate between sRAGE and fl/mRAGE, it is difficult to be certain which form is being detected in the cytoplasm. However increased membrane bound fl/mRAGE was seen in all CF patients and to a lesser extent in CFRD and diabetics, but was totally absent in healthy individuals. This shift in fl/mRAGE localisation represents a greater risk of disease promotion, as fl/mRAGE can be easily activated by RAGE ligands particularly when associated with low sRAGE.

Amongst the specific ligands for RAGE are AGEs, a heterogeneous class of compounds produced by glycation and oxidation of proteins and lipids. Many S100 calcium binding proteins also bind RAGE including enRAGE that binds only to RAGE. This is the first study to assess enRAGE and AGE levels in induced sputum in healthy volunteers, diabetics, CF and CFRD. However it is inappropriate to look at them in isolation; it is more logical to assess them relative to both sRAGE and membrane bound fl/mRAGE. The higher enRAGE/sRAGE ratio in CFRD patients compared to diabetics and healthy subjects is almost certainly impacting on the pro-inflammatory environment of the CF-diabetic airway. In the blood compartment of the diabetic patients there was an increase in enRAGE/sRAGE and AGE/sRAGE ratios supporting this being a useful index for monitoring inflammatory activity in diabetics. Determining the ratio of sRAGE to mRAGE would also be useful but currently is difficult to quantify.

Our study highlights the need for a complex analysis of data when investigating the role of RAGE in disease. Diabetes is a systemic disease and serum levels appear to be relevant while in contrast, measurements of serum RAGE appeared to have little merit in CF patients. Although we have shown that sputa data appears to be reflective of airway disease, it is a challenge to evaluate the relative impact of one component of the RAGE system over another and the interactions occurring. Therefore in assessing the sputa data we integrated all of the RAGE components in our analysis. This is quite different to previous studies which have simply assessed components in isolation. There were clear differences between patient groups. CF patients were unmistakably identifiable from healthy and diabetic patients as demonstrated in Figures 6 and 7. The ratio between enRAGE/sRAGE and AGE/sRAGE in sputa strongly supports RAGE contributing to the increased lung deterioration seen in the CFRD group, despite single mediator analysis not demonstrating this. This data questions the merit of only measuring AGE and enRAGE levels rather than their ratios with sRAGE.

This was a cross sectional study and this created its own limitations in terms of interpreting RAGE dynamics in patients. Future studies should aim to assess RAGE expression in patients when stable and unwell and over a number of time points, but this was outside the scope of the current study.

The role of RAGE in CF and CFRD is complex. This is a pilot study and describes the differences in the RAGE pathway in blood and sputa in CF with and without diabetes, in diabetics and in healthy subjects and establishes that an association between RAGE related inflammation and the inflammatory overdrive seen in CF lung disease is likely. Alterations in the expression of key mediators of the RAGE pathway specific to CFRD were demonstrated and represent a plausible explanation for the deterioration in lung function seen in CFRD. This study uses a complex, multifaceted approach to assess the role of RAGE in CF that is applicable to other chronic airway diseases and helps identify the respective contributions of protective sRAGE, membranous RAGE and RAGE ligands on airway inflammation and their capacity to act as disease biomarkers. Further assessment of RAGE activation will provide greater clarity as to the relative importance of the various components of the RAGE system and open up the potential for new therapies.

## Methods

All methods described within the manuscript complied with the MIBBI guidelines where appropriate.

### Patient recruitment

The study was approved by the Human Research Ethics committee of Sir Charles Gairdner Hospital (SCGH). Patients were recruited randomly from the SCGH adult CF clinic, SCGH diabetes clinic and the Lung Institute of Western Australia Clinical Trials Unit. Written informed consent was obtained from all participants and the methods carried out complied with the approved guidelines.

### Sputum induction, processing and immunolabelling for RAGE receptor

Spontaneous sputum was collected from CF and CFRD patients and sputum induction performed on diabetic and healthy individuals. Sputum induction followed a standard protocol^19^, with a 4.5% saline solution inhaled for a total of 15.5 min from an ultrasonic nebulizer (Ultraneb 2000, PA, USA) after measurement of baseline FEV_1_. FEV_1_ was measured at 30 sec, 1 min, 2 min, and three × 4 min and subjects were encouraged to expectorate sputum.

All collected sputum samples were solubilised with 4 vol. of 0.1 % dithiothreitol and after centrifugation (400 g, 10 min), the cell-free supernatant was stored at −80°C for RAGE assay analysis. The cell pellet was resuspended in phosphate-buffered saline (PBS) and the total cell count and cell viability was assessed. Cytospins were prepared and stored in the dark at room temperature. Cytospins were fixed in acetone-methanol (1:1). After rehydration (0.01 M PBS, pH 7.4) peroxidase activity was inhibited with peroxidase block *(DAKO, Sydney, Australia)* for 5 min. Non-specific protein binding was blocked with 20% swine serum and serum-free protein block *(DAKO, Sydney, Australia)* for 15 min each. Slides were then incubated with RAGE antibody *(Abcam, Cambridge, UK)* at a dilution of 1/100 in 0.01 M PBS containing 1% bovine serum albumin (BSA) overnight at room temperature following incubation with anti-rabbit horseradish peroxidase conjugated polymer (*DAKO, Sydney, Australia)* for 30 min at room temperature. Labelling was visualized by incubating the slides with 3,3′-diaminobenzidine (DAB), counter staining with Mayer's haematoxylin and differential white cell staining (Diff-Quick stain). The specificity of immuno-labelling was verified by negative control slides from which the primary antibody was omitted.

Assessment of sputum differential cell count and flRAGE expression staining was undertaken. Staining was assessed by two observers independently. A positive result was assigned when diffusely cytoplasmic or strong membranous staining was observed in leukocytes. Staining intensity was graded semi-quantitatively based on Remmele's sliding scale: negative (0), weak (1), moderate (2) and strong (3)^20^ Intensity values of scores 2 and 3 were considered to represent positive flRAGE expression. In discrepant cases a consensus opinion was reached.

### Separation of peripheral blood leukocytes

Whole blood was collected in EDTA and passed through LeukoLOCK filters *(LeukoLOCK Total RNA Isolation System, Ambion Inc. USA)*. The filters captured the total leukocyte population, while plasma, platelets and red blood cells were eliminated. The filters were flushed with PBS to remove residual red blood cells and then with RNAlater (*LeukoLOCK)* to stabilize leukocyte RNA. Filters were sealed and stored at -80°C until further processing.

### RNA isolation

LeukoLOCK filters were thawed to RT for 5 min and flushed with TRIzol Reagent (*Life Technologies, Australia*). RNA was separated into aqueous phase by chloroform and total RNA isolated using PureLink RNA Mini Kit *(Life Technologies*). Total RNA from sputum cells was extracted using RNeasy Mini kit (*Qiagen, Australia*).

### Real time PCR

Fl/mRAGE and esRAGE mRNA expression in blood and sputum were assessed by quantitative real-time PCR (*StepOnePlus, Applied Biosystems, Australia*) using flRAGE (HS00542592_G1) and soluble esRAGE (HS00542584_G1) specific primer-probes (*Applied Biosystems*), using the QuantiTect primer-probe One Step RT PCR kit (*Qiagen*). Cycle conditions were 50°C for 20 min; 95°C for 15 min; 45 cycles of 95°C for 1s and 60°C for 1 min. Each sample was run in triplicate and relative expression levels calculated by normalising the targets to the endogenously expressed housekeeping gene human peptidylpropyl isomerase A.

### Elisa

Concentrations of RAGE were determined in serum and sputum using ELISA kits sRAGE (*R&D Systems, USA*), esRAGE (*B-Bridge International, USA*) and the RAGE ligands: enRAGE (*CircuLex, USA*) and AGEs (*Cell Biolab, USA*), using standard protocols.

### Statistical analysis

Data are presented as mean ± Standard Error of the Mean (SEM). GraphPad Prism (*San Diego, USA*) was used for data analysis. ANOVA followed by multiple comparisons testing using Tukey's test was used to determine significant differences between group means. In all instances, a *P* value less than 0.05 was considered significant.

## Author Contributions

S.M. and S.B. contributed to the study conception, study design and drafting of article. S.B., S.A., A.M. and F.F. contributed to acquisition of data and analysis and interpretation of data. S.A., S.M., J.W. and J.K. contributed to patient recruitment. P.J.T. contributed to the study conception, study design, interpretation of data and critically revised the article. All authors approved the final version of article to be published.

## Figures and Tables

**Table 1:** Clinical Parameters of recruited patients

Characteristic	Healthy	CF	CFRD	Diabetic	*P*
Gender (M/F)	4/6 (n = 10)	4/1 (n = 5)	2/3 (n = 5)	5/2 (n = 7)	-
Age (Years)	38.9 ± 9.47	31.4 ± 9.71	33.6 ± 10.88	25.29 ± 7.76	0.1079
FEV1/FVC % predicted	81.60 ± 8.51	62.59 ± 17.99*	60.39 ± 9.47*	92.14 ± 13.03	0.0002*
BMI (kgm^−2^)	25.5 ± 4.06	21.61 ± 3.58	30.72 ± 21.13	27.46 ± 6.01	0.5182
HbA1c (%)	-	5.9 ± 0.22	6.98 ± 0.75*	7.57 ± 0.93*	0.0282*0.0072*
CRP	-	12.97 ± 4.63	4.57 ± 3.32	-	0.063
WCC	-	9.57 ± 1.86	10.9 ± 2.96	-	0.5277

The clinical parameters are shown as mean ± standard deviation, *statistically significant; M, Male; F, Female; CF, cystic fibrosis; CFRD, cystic fibrosis related diabetes; FEV1/FVC, forced expiratory volume/forced vital capacity; BMI, body mass index; HbA1c, haemoglobin A1c; CRP, C-reactive protein; WCC, white blood cell count.
